# Use of a Sec signal peptide library from *Bacillus subtilis* for the optimization of cutinase secretion in *Corynebacterium glutamicum*

**DOI:** 10.1186/s12934-016-0604-6

**Published:** 2016-12-07

**Authors:** Johannes Hemmerich, Peter Rohe, Britta Kleine, Sarah Jurischka, Wolfgang Wiechert, Roland Freudl, Marco Oldiges

**Affiliations:** 1 Institute of Bio- and Geosciences-Biotechnology (IBG-1), Forschungszentrum Jülich, Jülich, Germany; 2 Institute of Biotechnology, RWTH Aachen University, Aachen, Germany; 3 Bioeconomy Science Center (BioSC), Jülich, Germany; 4 Boehringer Ingelheim Pharma GmbH and Co. KG, Biberach, Germany; 5 Thermo Fisher Scientific GENEART GmbH, Regensburg, Germany

**Keywords:** *Corynebacterium glutamicum*, Industrial enzyme production, Protein secretion, Sec signal peptide library, Microbioreactor, Mini-Pilot-Plant

## Abstract

**Background:**

Technical bulk enzymes represent a huge market, and the extracellular production of such enzymes is favorable due to lowered cost for product recovery. Protein secretion can be achieved via general secretion (Sec) pathway. Specific sequences, signal peptides (SPs), are necessary to direct the target protein into the translocation machinery. For example, >150 Sec-specific SPs have been identified for *Bacillus subtilis* alone. As the best SP for a target protein of choice cannot be predicted a priori, screening of homologous SPs has been shown to be a powerful tool for different expression organisms. While SP libraries between closely related species were successfully applied to optimize recombinant protein secretion, this was not investigated for distantly related species. Therefore, in this study a Sec SP library from low-GC firmicutes *B. subtilis* is investigated to optimize protein secretion in high-GC actinobacterium *Corynebacterium glutamicum* using cutinase from *Fusarium solani pisi* as model protein.

**Results:**

A homologous SP library (~150 SP) for recombinant cutinase secretion in *B.* *subtilis* was successfully transferred to *C.* *glutamicum* as alternative secretion host. Cutinase secretion in *C.* *glutamicum* was quantified using an automated micro scale cultivation system for online growth monitoring, cell separation and cutinase activity determination. Secretion phenotyping results were correlated to those from a previous study, in which the same SP library was used to optimize secretion of the same cutinase but using *B. subtilis* as host. Strikingly, behavior of specific SP-cutinase combinations was changed dramatically between *B.* *subtilis* and *C.* *glutamicum*. Some SPs showed comparable cutinase secretion performances in both hosts, whereas other SPs caused diametrical extracellular cutinase activities.

**Conclusion:**

The optimal production strain for a specific target protein of choice still cannot be designed in silico. Not only the best SP for a target protein has to be evaluated each time from scratch, the expression host also affects which SP is best. Thus, (heterologous) SP library screening using high-throughput methods is considered to be crucial to construct an optimal production strain for a target protein.

**Electronic supplementary material:**

The online version of this article (doi:10.1186/s12934-016-0604-6) contains supplementary material, which is available to authorized users.

## Background

A major part of industrial biotechnology is the production of technical bulk enzymes, with an estimated market of 1 billion US-$ in 2010 [1]. To supply this market with sufficient quantities and in an economically feasible manner, platform technologies are developed continuously. In this context, expression hosts that allow the secretory production of proteins are preferred. With extracellular, secretory enzyme formation, product recovery is facilitated because time- and energy-consuming cell disruption and downstream processing are avoided. This is reflected by the fact that dominating expression hosts for the production of commercialized enzymes are secretory hosts, like *Aspergillus* sp. and *Bacillus* sp. with a share of about 27 and 17%, respectively [2], representing almost half of total enzyme production.

### *Corynebacterium glutamicum* as host organism for protein production

For the production of technical bulk enzymes, different Gram-positive expression hosts are available. These mostly monoderm bacteria are robust in terms of cultivation conditions, low nutritional demand, and are able to secrete proteins into the extracellular medium [3, 4]. Next to *Bacillus* sp., a powerful expression host for the production of technical enzymes [5], *Corynebacterium glutamicum* is an attractive alternative host microorganism with demonstrated protein secretion capacity in the g/L-range [6, 7]. Also, extensive bioprocess knowledge with *C.* *glutamicum* and methods for genetic manipulation are available because this microbe is a major producer for amino acids at industrial scale for decades [8]. Recently, the construction of a *C.* *glutamicum* strain harboring the DE3/T7 expression system was reported, allowing to control gene expression levels for intracellular protein production [9]. This system is based on the prophage-cured *C.* *glutamicum* strain MB001, which was shown earlier to be beneficial for intracellular protein production [10]. Besides, *C.* *glutamicum* strain ATCC13869 was commercialized as protein expression system under the trademark “CORYNEX” by the Japanese company Ajinomoto. This system was recently improved for secretory antibody Fab fragment production by deletion of *cspB* and *pbp1a* [11].

### General secretory pathway

Extracellular protein production in Gram-positive expression hosts like *B.* *subtilis* or *C.* *glutamicum* is mostly achieved by employing the highly conserved general secretory (Sec) pathway. For example, in *B.* *subtilis* most naturally secreted proteins are Sec substrates [12]. Proteins to be secreted are marked by an upstream sequence, the signal peptide (SP), which is ~30 amino acids in length on average [12]. These SPs show a highly conserved three-domain structure: (1) a positively charged N-region, with a high preference for lysine residues at P2 and P3 [12, 13], (2) the longest region, termed H-region, consists of hydrophobic amino acids, and (3) a C-domain, which contains a cleavage site (typical motif: A-X-A) between the signal peptide and target (pre-) protein to be recognized by signal peptidase (SPase). In contrast to the highly conserved three-domain structure of SPs, a high diversity in amino acid composition within these domains is observed. Proteins marked by a Sec SP are recognized by the signal recognition particle (SRP), transported to the Sec translocon in the cytoplasmic membrane and ultimately translocated in an unfolded manner by the SecA protein through SecYEG which forms the actual protein translocation pore spanning the cytoplasmic membrane. The SP itself is cleaved at the trans-side of the membrane by SPase, after which the translocated protein finally folds into its functional conformation [14]. SecD and SecF assist protein translocation by a pulling force directed to the trans-side of the membrane fueled by proton motive force [15].

Besides, proteins can be translocated via TAT pathway into which the target protein is directed by fusion to a TAT-specific SP containing two arginine residues (TAT = “twin arginine translocation”). In contrast to the Sec pathway, proteins gain their mature conformation in the cytoplasm before being translocated by the TAT machinery [14].

### Signal peptide screening for optimized secretion of heterologous proteins

In several studies, the SP has been shown to be a critical factor for recombinant protein secretion. From the genome of *B.* *subtilis*, 173 Sec SPs have been identified based on the highly conserved three-domain structure [12], which have been compiled by Brockmeier et al. into a genetic library for optimizing recombinant protein secretion in *B.* *subtilis* [16]. The study revealed that systematic screening of all natural SPs in *B.* *subtilis* is a powerful strategy to enhance extracellular enzymatic activity using cutinase from *Fusarium solani pisi* as model enzyme. The SP was found to affect the processing kinetics of cutinase preprotein during translocation, but a fast or low cutinase preprotein processing could not be correlated to high or low extracellular cutinase activities, respectively. Furthermore, the SP screening results for cutinase could not be transferred to a second model enzyme, an esterase from metagenomics origin.

In a follow-up study by Caspers et al. [17], a closer look at the N-domain of the AmyE-SP from *B.* *subtilis* by saturation mutagenesis demonstrated a high potential for optimizing extracellular protein production via SP variation, also with cutinase as model enzyme (from 20% up to 400%). The different obtained mutants resulted in different processing kinetics of cutinase precursor, but again these could not be correlated to the amount of extracellular cutinase activity [17].

Degering et al. conducted another study to optimize recombinant enzyme secretion with *B.* *subtilis* by SP library screening [18]. The SP library from *B. subtilis* introduced by Brockmeier et al. [16] was extended by the natural SPs from *B.* *licheniformis*. This resulted in a mixed library with ~400 SPs, which was screened for the secretion of subtilisin BPN’ from *B.* *amyloliquefaciens* as model enzyme using *B.* *subtilis* as expression host. It was found that maximal extracellular subtilisin BPN’ activity in *B.* *subtilis* was achieved with a heterologous SP from *B.* *licheniformis*. Moreover, the eight best performing SPs yielded considerably higher extracellular subtilisin BPN’ activities (~750 to ~350%) compared to the native SP from *B.* *amyloliquefaciens*. In a second step, these eight best performing combinations of SP and subtilisin BPN’ were transferred into two *B.* *licheniformis* strains for expression. It was found that the results were comparable between *B.* *subtilis* and the two *B.* *licheniformis* strains, and highly comparable between the two *B.* *licheniformis* strains.

For another Gram-positive expression host, *Lactobacillus planatarum*, screening of homologous SPs was applied by Mathiesen et al. [19] to optimize secretory production using staphylococcal nuclease (NucA) and lactobacillal amylase (AmyA) as model enzymes. As seen in other studies, no correlation of SP performance between different target proteins could be observed, i.e. NucA and AmyA [19].

Watanabe et al. browsed the genome of *C.* *glutamicum* R for predicted SPs, which were then screened for secretion of α-amylase from *Geobacillus stearothermophilus* [20]. Several of these SPs were shown to outperform the well-known corynebacterial PS2 SP with respect to extracellular amylase activity, highlighting again that for each target protein the optimal SP has to be identified for maximal secretion efficiency.

Recently, Zhang et al. [21] investigated 114 Sec SPs from *B.* *subtilis* for secretion of an alkaline xylanase from *Bacillus* *pumilus* BYG using *B.* *subtilis* as expression host [21]. Two promoters of different strength (P43 and P*glvm*) were compared for xylanase secretion with the SP library. The comparison of the secretion efficiencies for the tested SP-xylanase combinations under control of either P43 or P*glvm* showed comparable results with a high correlation for these two promotors. This indicates that transcript level of SP-xylanase fusion is of minor importance to the secretion efficiency measured as extracellular xylanase activity. Clearly, the signal peptide was the major factor for xylanase secretion.

In summary, several studies revealed that recombinant protein secretion in different hosts can be adjusted over a wide range by screening endogenous Sec SPs. In addition, the transfer of SPs within different *Bacillus* sp. offers great potential for further enhancement of protein secretion. As seen in previous studies, Sec SPs from low-GC firmicutes *B.* *subtilis* are functional in high-GC actinobacterium *C.* *glutamicum* [22] and therefore, the assessment of the Sec SP library introduced by Brockmeier et al. [16] seems to be a promising approach to optimize recombinant protein secretion also in *C.* *glutamicum*.

## Methods

### Bacterial strains, media, and growth conditions

*Bacillus subtilis* TEB1030 (*his nprE aprE bpf ispI lipA lipB*) [23] and *Escherichia coli* JM109 (*e14* ^−^ *(McrA* ^−^ *) recA1 endA1 gyrA96 thi*-*1 hdsR17(r*_*K*_^−^ *m*_*K*_^+^ *) supE44 relA*) (Stratagene, Heidelberg/DE) were grown at 37 °C in LB medium containing 10 g/L tryptone, 10 g/L yeast extract, and 5 g/L NaCl, supplemented either with 100 mg/L ampicillin (*E.* *coli*) or 25 mg/L kanamycin (*B.* *subtilis*), respectively. *Corynebacterium glutamicum* ATCC13032 [24] was grown in BHI medium containing 37 g/L brain heart infusion (Difco), BHIS medium containing 37 g/L brain heart infusion and 91 g/L sorbitol, or CgXII minimal medium [25] containing 20 g/L glucose, 20 g/L (NH_4_)_2_SO_4_, 5 g/L urea, 1 g/L KH_2_PO_4_, 1 g/L K_2_HPO_4_, 13.25 mg/L CaCl_2_ · 2 H_2_O, 0.25 g/L MgSO_4_ · 7 H_2_O, 0.2 mg/L biotin, 30 mg/L protocatechuic acid (PCA), 10 mg/L FeSO_4_ · 7 H_2_O, 10 mg/L MnSO_4_ · H_2_O, 1 mg/L ZnSO_4_ · 7 H_2_O, 0.313 mg/L CuSO_4_ · 5 H_2_O, 0.02 mg/L NiCl_2_ · 6 H_2_O. As buffering agent, 42 g/L MOPS was added and pH was adjusted to 7.0 using 4 M NaOH. For maintaining selection pressure, 25 mg/L kanamycin or 30 mg/L chloramphenicol was added. If required, isopropyl-ß-d-thiogalactopyranoside (IPTG) was added at a concentration of 500 µM. All chemicals were of analytical grade and supplied by Sigma Aldrich.

### Plasmid constructions

Routine methods such as DNA isolation, restriction and ligation were performed using standard protocols [26]. The correctness of all newly constructed plasmids was verified by DNA sequencing. 148 individual glycerol stocks of *B.* *subtilis* cells, each containing a different pBSMuL3-SP-cutinase plasmid that encodes one of 148 SPs from *B.* *subtilis* fused to the cutinase from *Fusarium solani pisi* [16], were plated out on LB agar plates to single colonies which were subsequently used to inoculate 5 mL LB medium. The 148 cultures were grown overnight at 37 °C to stationary phase. Subsequently, the 148 cultures were combined and a plasmid mixture (pBSMul3-SP^Lib^-cutinase) encoding the SP library fused to cutinase was isolated from the respective mixed overnight cultures. To allow expression in *C.* *glutamicum*, pBSMul3-SP^Lib^-cutinase was digested with *Hind*III/*Bam*HI and the DNA fragment containing the SP^Lib^-cutinase encoding genes was ligated into the *Hind*III/*Bam*HI-digested *C.* *glutamicum* expression vector pXMJ19 [27]. In the resulting plasmid mixture pXMJ19-SP^Lib^-cutinase, the respective SP-cutinase genes are placed under the regulatory control of the IPTG-inducible P_tac_ promoter. The cloning of a selection of genes encoding four SPs from *B.* *subtilis* (AmyE, NprE, YpjP, YwmC) fused to cutinase into the *C.* *glutamicum* expression vector pEKEx2 [28] has been reported previously [22]. *C.* *glutamicum* cells were transformed by electroporation as described [29].

### Micro scale cultivation in robotic environment (Mini-Pilot-Plant)

Precultures were grown in standard 96 well microplates (Greiner, Frickenhausen/DE) sealed with gas permeable membranes. Medium volume was 200 µL containing appropriate antibiotics at 30 °C. The microplates were shaken at 900 rpm at a shaking diameter of 1.5 mm using a bench-top device (“Titramax 100”, Biotest, Dreieich/DE) placed in temperature-controlled (30 °C) cabinet (Edmund Bühler, Hechingen/DE). Precultures were inoculated from single colonies formed after transformation of pXMJ19-SP^Lib^-cutinase into *C.* *glutamicum* (see above). After 8 h, 50 µL of the precultures were used to inoculate main cultivations that were grown in baffled 48 well microtiter plates sealed with gas permeable membranes (“Flowerplate”) in a microbioreactor (“BioLector”) cultivation device (m2p-labs, Baesweiler/DE). Cultivation conditions were as follows: CgXII medium, working volume of 1000 µL, orbital shaking with 1200 rpm at a shaking diameter of 3 mm. The microbioreactor is integrated into a liquid handling robot (PerkinElmer, Waltham, MA/USA), as described earlier [22] and referred to as “Mini-Pilot-Plant” (MPP) [30].

The online monitored biomass concentration via backscatter (BS) measurement served as trigger signal for automated culture induction and harvest. In the case of *C. glutamicum*, IPTG was added to a final concentration of 500 µM when a BS value corresponding to 4 g/L cell dry weight was reached. After further 4 h later, the culture was harvested and stored in 96 deep well plates (“Riplate”, Ritter, Schwabmünchen/DE), which were placed on cooling carriers held at 4 °C. Main cultivations of *B.* *subtilis* were programmed to be harvested and stored likewise 1 h after reaching a BS value corresponding to 1.17 g/L cell dry weight. Cultivations of *B.* *subtilis* were not induced due to constitutive expression using the pBSMuL3 system. After all individual cultures of a main cultivation have been harvested, the cooled cell suspensions were clarified by centrifugation using a robot accessible centrifuge (“IXION”, Sias, Hombrechtikon/CH). After centrifugation at 4000*g* for 15 min, the supernatant was obtained and used for determination of cutinase activity.

### Cutinase activity assay

Determination of cutinase activity in cultivation supernatants was performed spectrophotometrically using p-Nitrophenylpalmitate (pNPP) as substrate analogon [31] as reported elsewhere [22]. Briefly, 20 µL of appropriately diluted supernatants were transferred to a standard 96 well microplate. 20 µL of water was applied as blank. The enzymatic reaction was started by rapid addition of 180 µL reaction solution with a multichannel pipette (“Research Pro 1200”, Eppendorf, Hamburg/DE). Reaction solution was freshly prepared by combining 9 volumes of 50 mM phosphate buffer pH 8 supplemented with 2.3 g/L Na-desoxycholate and 1.11 g/L gum arabic, with 1 volume of 30 mg pNPP in 10 mL 2-propanol. Immediately after addition of the reaction solution, the microplate was transferred to a microplate reader pre-heated to 37 °C and absorption at 410 nm was measured each 25 s. The obtained linear slope was blanked and cutinase activity in kU/L was calculated using a molar extinction coefficient (15 cm^2^/µmol).

## Results and discussion

### Dynamic harvest procedure reproduces signal peptide screening results from cutinase secreting *B.* *subtilis* strains with lower statistical error

**Fig. 1:**
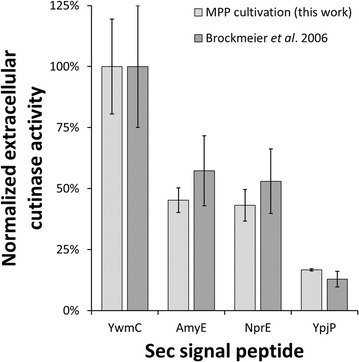
Comparison of extracellular cutinase activities using *B. subtilis* pBSMuL3-SP-cutinase as expression host with different Sec signal peptides. Cutinase activities reported by Brockmeier et al. [16] are reproduced using the MPP cultivation setup, but with lower statistical error. Cutinase activities are normalized by the maximal value of the corresponding data series, error bars as standard deviation from eight biological replicates (this work) or 25% as reported by Brockmeier et al. [16]

In this study, an integrated system of microbioreactor and liquid handling robot [22] was applied for evaluation of SP performance with respect to the secretory production of cutinase using *C.* *glutamicum*. To allow a direct comparison of the results for SP impact on cutinase secretion with *B.* *subtilis* obtained by Brockmeier et al. [16], four *B.* *subtilis* expression strains with different signal peptides (YwmC, AmyE, NprE and YpjP) were re-assessed using the MPP cultivation and harvest setup. As seen in Fig. 1, the expression strains with the different signal peptides employed are classified according to the extracellular cutinase activity, and this classification found by Brockmeier et al. [16] is maintained for the MPP cultivation. However, for the MPP cultivation a lower statistical error is found.

Whereas Brockmeier et al. [16] employed 96 deep well blocks (DWP, 2000 µL total well volume) with a working volume of 1000 µL and a shaking frequency of 600 rpm at a shaking diameter of 3 mm, this study uses flower-shaped 48 well microplates for cultivation with 1000 µL and 1200 rpm at 3 mm. Briefly, these parameters are effectors of the maximal oxygen transfer capacity, i.e. the availability of oxygen to the culture [32]. As Brockmeier et al. [16] used DWP without oxygen sensitive fluorescence sensor spots (optodes) to monitor dissolved oxygen (DO) of the cultures, oxygen limited growth cannot be excluded. Furthermore, different harvest strategies are used: The online-monitoring of biomass in this study allows to determine end of growth for each culture individually, a feature Brockmeier et al. [16] had not at hand and thus, all cultures were harvested after a fixed time of 16 h. The reproducible detection of growth phases using the MPP and the resulting reproducible harvest procedure just at the end of the exponential growth provides an explanation for the observed lower statistical error [22].

The results indicate the technically more sophisticated MPP cultivation and harvest setup does not change the classification of the results obtained by Brockmeier et al. [16], which is an important prerequisite to compare SP screening results for cutinase secretion between *C.* *glutamicum* and *B.* *subtilis* as expression host. Clearly, the lower statistical error due to more precise harvest procedure allows for more precise differentiation of SPs with similar extracellular cutinase activities.

### The secretion phenotype of randomly selected *C. glutamicum* clones can be reproducibly classified based on extracellular cutinase activity

The presented MPP cultivation and harvest workflow starts with single colonies formed after plating a transformation mix of *C.* *glutamicum* cells. These colonies serve as inoculation material for the precultures grown in complex BHI medium supporting fast growth to generate a sufficient amount of cell mass. These cultures then are used as inoculation material for the MPP cultivations with induced cutinase secretion in defined CgXII minimal medium.

This workflow, beginning with preculturing from a single colony after library transformation needs to be validated. Hence, a mixture of *C.* *glutamicum* cells harboring different expression plasmids as encountered after library transformation (cf. “Plasmid constructions” section) was simulated by a mixture of deep frozen cryo stock aliquots of isogenic cells carrying either the AmyE, NprE, YpjP or YwmC SP for pEKEx2-based cutinase secretion. A strain with the empty vector as control was also included. The resulting mixed cell suspension, differing in the vector insert, was plated and incubated until single colonies appeared. Then, 48 colonies were picked randomly for preculture inoculation with subsequent MPP cultivation and harvest as described above. For comparison, the different expression strains were plated individually and from each of these plates, eight colonies (with known expression plasmid) were picked and treated likewise.

**Fig. 2:**
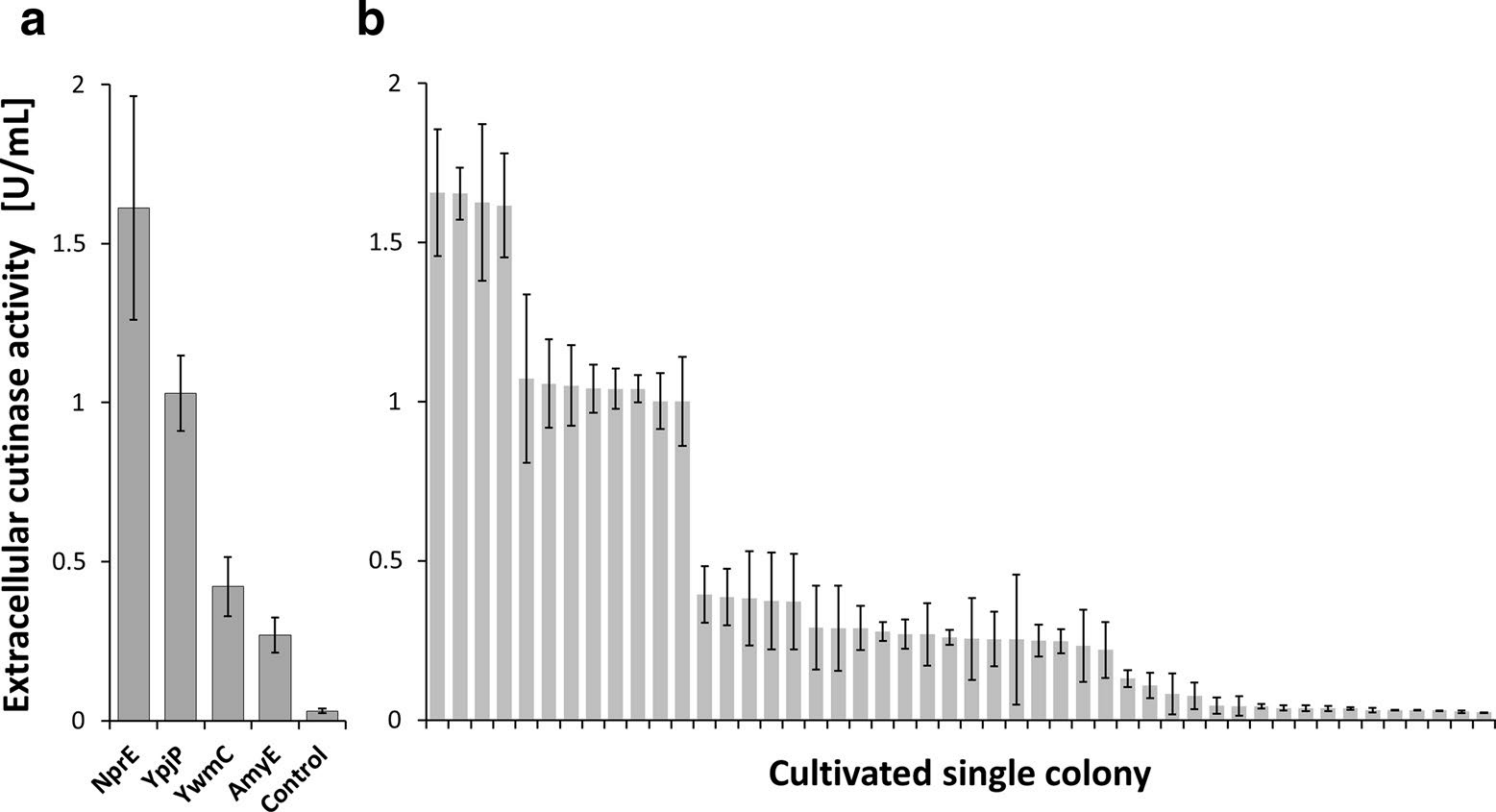
Identification of *C.* *glutamicum* strains with different signal peptides for cutinase secretion based on extracellular cutinase activities. **a** Cutinase activities obtained from MPP cultivations using single *C.* *glutamicum* colonies with defined signal peptide insert for inoculation. Colonies were plated from a frozen cryostock of the corresponding expression strain. *Error bars* indicate standard deviation from eight replicates. **b** Likewise, but for obtaining agar colonies a mixture of the frozen cryostocks was plated yielding single colonies with undefined signal peptide insert for cutinase secretion. From these, 48 colonies were randomly picked as inoculation material for 48 MPP cultivations. *Error bars* indicate standard deviations from three cutinase measurements

Figure 2a depicts the SPs used for cutinase secretion from the reference cultivations, ordered by the resulting extracellular cutinase activity. In comparison, Fig. 2b shows the 48 cultivations of *C.* *glutamicum* with undetermined expression plasmids, ordered likewise. The randomly selected clones can be classified to carry NprE-cutinase, YpjP-cutinase or the other inserts based on the comparison of found cutinase activities with those from reference cultivations (detailed results are given in the Additional file 1). Clones with undetermined SPs suspected to be YwmC or AmyE (Fig. 2b) cannot be identified unambiguously according to their respective cutinase activity, as seen also for the reference cultivations (Fig. 2a). However, the procedure identifies the best SP for cutinase secretion safely, namely the NprE SP. In case one is interested in the identification of expression plasmids yielding indistinguishable secretion phenotypes, plasmid extraction and sequencing needs to be performed.

The results indicate that the selection of single colonies after transformation of a genetic library, i.e. the transformation with a mixture of plasmids, results in consistent recovery of a specific phenotype with its connected specific genotype, i.e. a specific expression strain. This is important, as the clonal selection after library transformation is the separation step for the library elements during the screening workflow. An alternative procedure would be to clone each SP-protein fusion into an expression vector with subsequent transformation. Such a procedure is characterized by an excessive workload because of necessary individual cloning and transformation due to the large library size, especially when combining several libraries.

### The relative efficiency of Sec signal peptides with respect to cutinase secretion differs between low-GC *B.* *subtilis* and high-GC *C.* *glutamicum*

**Fig. 3:**
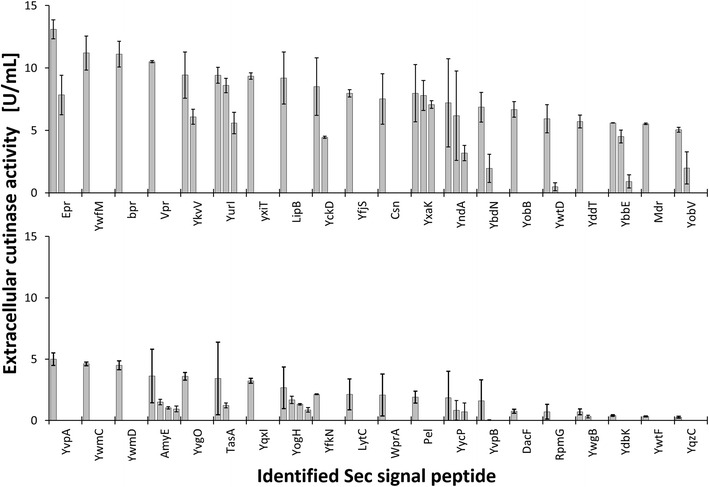
Identified Sec SP from *C.* *glutamicum* pXMJ19-SP^Lib^-cutinase MPP cultivations with corresponding extracellular cutinase activities. Cultivations were inoculated from single colonies randomly selected after transformation of the SP library. *Multiple bars* indicate SPs that have been identified several times. *Error bars* represent standard deviations from two cutinase measurements

After cloning the Sec SP library from pBSMuL3-SP^Lib^-cutinase to pXMJ19-SP^Lib^-cutinase as described in “plasmid construction ” section, pXMJ19-SP^Lib^-cutinase was transformed into *C.* *glutamicum* and the transformation mix was plated and incubated until appearance of single colonies. From these, 66 have been selected randomly for characterization by MPP cultivation and harvest with subsequent determination of extracellular cutinase activity. Afterwards, the expression plasmids were extracted from the individual cultures and sequenced for identification of the inserted SP-cutinase fusion. Results are depicted in Fig. 3.

Most of the signal peptides have been identified once, some twice and only a few three or four times. When treating the screening procedure as Bernoulli process, the probabilities to hit a SP once, twice, three or four times are calculated to be 0.287, 0.064, 0.009 or 0.001, respectively. The experimentally found relative occurrences of SPs identified multiple times are somewhat higher (0.379, 0.136, 0.076 or 0.030, respectively), but in good agreement with the theoretical values. A simulation of the clone screening procedure found also number ranges of multiple SP occurrences comparable to the empirically determined values (cf. Additional file 1). Thus, it is assumed that the library could be transferred completely from *B.* *subtilis* to *C.* *glutamicum*, so that all SPs contained in the *B.* *subtilis* derived SP library had the same probability to be cloned into the expression plasmid for *C.* *glutamicum*.

It can be seen that cutinase activities vary over a wide range depending on the SP used to target secretion of cutinase in *C.* *glutamicum*. This observation was made previously when using *B.* *subtilis* as expression host [16] and when screening homologous SP libraries for different target proteins [18–21]. This indicates that Sec SP from low-GC *B.* *subtilis* are functional in high-GC *C.* *glutamicum* in general. Therefore, the heterologous SP library from *B.* *subtilis* can be applied to optimize recombinant protein secretion in *C.* *glutamicum*. Moreover, screening of even more SPs in libraries, e.g. introduced by Degering et al. [18] or Watanabe et al. [20], is promising. Once an optimal combination of SP and target protein has been identified, a mutagenesis of the optimal SP [17] seems reasonable to further enhance protein secretion.

**Fig. 4:**
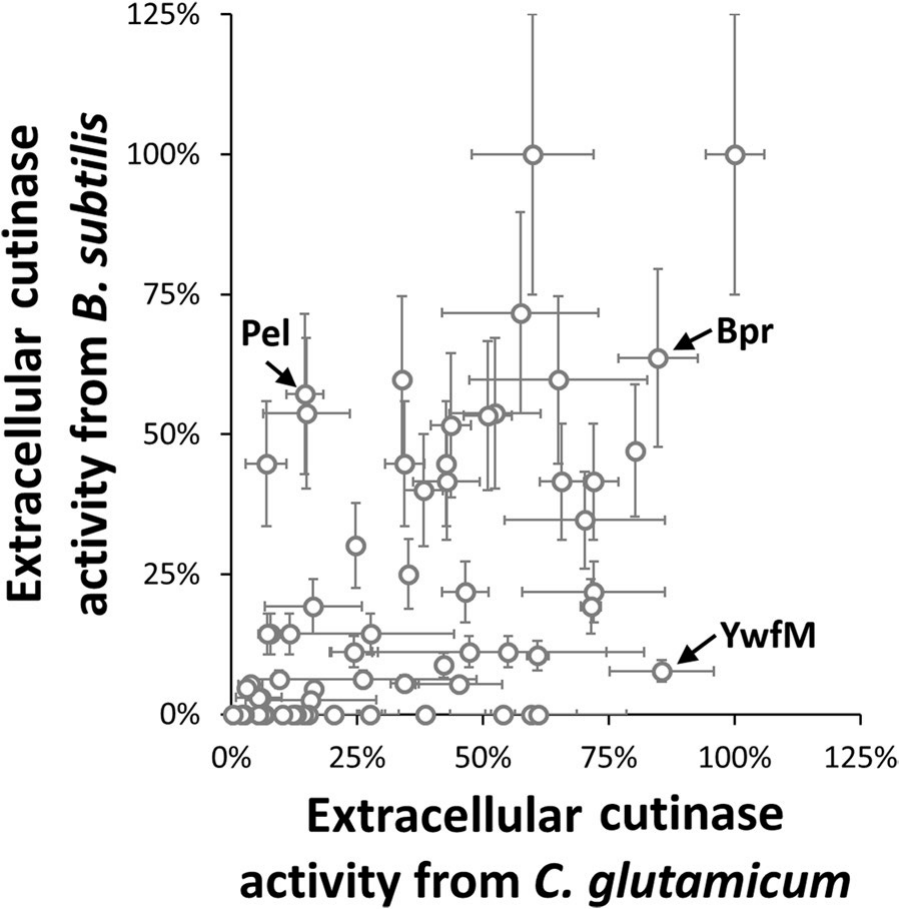
Comparison of signal peptide performance with respect to extracellular cutinase activities using *B.* *subtilis* pBSMuL3-SP^Lib^-cutinase or *C.* *glutamicum* pXMJ19-SP^Lib^-cutinase as expression host. Each data point represents one SP with its corresponding cutinase activity obtained from cutinase secretion in *B. subtilis* [16] or *C.* *glutamicum* (this study). Three marked SPs (Bpr, Pel, YwfM) with their corresponding extracellular cutinase activities in both secretion hosts are discussed in the text, cf. also Table 1. Both data series are normalized by their respective maximum value and error bars represent standard deviations (25% as reported for *B.* *subtilis* [16] or from two cutinase activity measurements for *C.* *glutamicum* in this study, respectively)

Degering et al. found that a performance ranking of specific SP-protease combinations with respect to extracellular protease activity was comparable when switching from *B.* *subtilis* to closely related *B.* *licheniformis* as secretion host, i.e. when switching from one species to another closely related species within the same genus [18]. Strikingly, this is not seen in this study where the SP library from *B.* *subtilis* was used in the distantly related *C.* *glutamicum*. In Fig. 4, extracellular cutinase activities in dependence of the specific SP are compared when using *B.* *subtilis* (data from [16]) or *C.* *glutamicum* as secretion host (this study). In contrast to the aforementioned findings by Degering et al. [18] no correlation is observed, indicating that the best SP for secretion of cutinase in *B.* *subtilis* is not the best SP for cutinase secretion in *C.* *glutamicum* and vice versa. Indeed, there are several SPs that perform well for cutinase secretion in *B.* *subtilis* and that perform very poorly in *C.* *glutamicum*. For other SPs the opposite situation is observed, and some SPs show comparable low or high cutinase secretion performance in both secretion hosts. For example, YwfM (~11.2 U/mL) and Bpr (~11.1 U/mL) yielded similar activities in *C.* *glutamicum*, but not in *B.* *subtilis* (YwfM: ~0.4 U/mL, Bpr: ~3.0 U/mL). On the other hand, Bpr (~3.0 U/mL) and Pel (~2.7 U/mL) yielded comparable activities in *B.* *subtilis*, but not in *C.* *glutamicum* (Bpr: ~11.1 U/mL, Pel: ~1.9 U/mL). This indicates that the behavior of specific SP-cutinase combinations is not predictable when distantly related organisms are used (cf. also Table 1 for detailed comparison of SPs).

**Table 1:** Comparison of extracellular cutinase activities depending on Sec signal peptide for *C.* *glutamicum* (this study) or *B.* *subtilis* (as reported by Brockmeier et al. [16])

Signal peptide	*C. glutamicum*		*B. subtilis*	
	Activity [U/mL]	Relative activity [%]	Activity [U/mL]	Relative activity [%]
Epr	13.1 ± 0.8	100.0 ± 5.8	4.7	100.0
*YwfM*	*11.2* ± *1.4*	*85.5* ± *10.4*	*0.4*	*7.7*
*Bpr*	*11.1* ± *1.0*	*84.8* ± *7.9*	*3.0*	*63.6*
Vpr	10.5 ± 0.1	80.2 ± 0.6	2.2	47.1
YkvV	9.4 ± 1.9	72.0 ± 14.1	1.0	21.8
YurI	9.4 ± 0.6	71.9 ± 4.9	1.9	41.5
YxiT	9.4 ± 0.3	71.4 ± 2.0	0.9	19.3
LipB	9.2 ± 2.1	70.2 ± 15.9	1.6	34.7
YurI	8.6 ± 0.6	65.6 ± 4.4	1.9	41.5
YckD	8.5 ± 2.3	64.9 ± 17.7	2.8	59.7
YfjS	8.0 ± 0.3	60.8 ± 2.2	0.5	10.5
Epr	7.8 ± 1.6	59.8 ± 12.1	4.7	100.0
YxaK	7.8 ± 1.2	59.5 ± 9.1	0.0	0.0
Csn	7.5 ± 2.0	57.4 ± 15.5	3.4	71.7
YxaK	8.0 ± 2.3	60.9 ± 17.5	0.0	0.0
YndA	7.2 ± 3.5	55.0 ± 27.0	0.5	11.1
YxaK	7.1 ± 0.3	54.0 ± 2.4	0.0	0.0
YbdN	6.9 ± 1.2	52.4 ± 9.1	2.5	53.7
YobB	6.7 ± 0.6	50.9 ± 4.8	2.5	53.3
YndA	6.2 ± 3.6	47.2 ± 27.3	0.5	11.1
YkvV	6.1 ± 0.6	46.5 ± 4.6	1.0	21.8
YurI	5.6 ± 0.9	42.7 ± 6.5	1.9	41.5
YwtD	5.9 ± 1.1	45.2 ± 8.6	0.3	5.4
YddT	5.7 ± 0.5	43.6 ± 4.0	2.4	51.6
YbbE	5.6 ± 0.0	42.7 ± 0.1	2.1	44.8
Mdr	5.5 ± 0.1	42.1 ± 0.5	0.4	8.8
YobV	5.0 ± 0.2	38.5 ± 1.5	0.0	0.0
YvpA	5.0 ± 0.5	38.2 ± 3.9	1.9	40.0
YwmC	4.6 ± 0.2	35.2 ± 1.2	1.2	25.1
YbbE	4.5 ± 0.5	34.4 ± 4.0	2.1	44.8
YwmD	4.5 ± 0.4	34.4 ± 2.8	0.3	5.6
YckD	4.4 ± 0.1	33.9 ± 0.7	2.8	59.7
YvgO	3.6 ± 0.3	27.5 ± 2.4	0.0	0.0
TasA	3.4 ± 3.0	26.1 ± 22.5	0.3	6.2
YqxI	3.2 ± 0.2	24.7 ± 1.4	1.4	30.2
YndA	3.2 ± 0.6	24.3 ± 4.7	0.5	11.1
YogH	2.7 ± 1.7	20.4 ± 12.9	0.0	0.0
YfkN	2.1 ± 0.0	16.3 ± 0.2	0.2	4.5
LytC	2.1 ± 1.3	16.2 ± 9.6	0.9	19.3
WprA	2.1 ± 1.7	15.8 ± 13.0	0.1	2.6
YobV	2.0 ± 1.3	15.2 ± 9.8	0.0	0.0
YbdN	1.9 ± 1.1	14.9 ± 8.6	2.5	53.7
*Pel*	*1.9* ± *0.5*	*14.5* ± *3.7*	*2.7*	*57.2*
YycP	1.9 ± 2.1	14.2 ± 16.3	0.0	0.0
YogH	1.7 ± 0.3	12.9 ± 2.3	0.0	0.0
YvpB	1.6 ± 1.7	12.2 ± 13.2	0.0	0.0
AmyE	1.5 ± 0.2	11.5 ± 1.8	0.7	14.3
YogH	1.3 ± 0.1	10.1 ± 0.5	0.0	0.0
TasA	1.2 ± 0.2	9.5 ± 1.5	0.3	6.2
AmyE	1.0 ± 0.1	7.8 ± 0.8	0.7	14.3
AmyE	0.9 ± 0.2	7.2 ± 1.8	0.7	14.3
YbbE	0.9 ± 0.5	6.9 ± 4.1	2.1	44.8
YogH	0.9 ± 0.2	6.6 ± 1.4	0.0	0.0
YycP	0.8 ± 0.8	6.3 ± 6.1	0.0	0.0
DacF	0.8 ± 0.2	5.8 ± 1.2	0.1	3.0
RpmG	0.7 ± 0.6	5.4 ± 4.6	0.1	3.0
YycP	0.7 ± 0.7	5.4 ± 5.6	0.0	0.0
YwgB	0.7 ± 0.2	5.3 ± 1.8	0.0	0.0
YwtD	0.5 ± 0.3	3.7 ± 2.4	0.3	5.4
YdbK	0.4 ± 0.1	3.0 ± 0.4	0.2	4.7
YwtF	0.3 ± 0.0	2.5 ± 0.3	0.0	0.0
YwgB	0.3 ± 0.1	2.5 ± 0.9	0.0	0.0
YqzC	0.3 ± 0.1	2.0 ± 0.5	0.0	0.0
YvpB	0.0 ± 0.0	0.3 ± 0.1	0.0	0.0

Signal peptides are sorted by their corresponding extracellular cutinase activity for *C.* *glutamicum*, multiple entries are found for Sec SPs that have been identified more than once. For these, the same cutinase activities reported by Brockmeier et al. have been assigned. Cutinase activities are given as absolute and relative values with respect to the maximal activity of the corresponding data series. Cutinase activity errors for *C. glutamicum* represent standard deviations from two analytical replicates. Cutinase activity errors for *B.* *subtilis* are 25%, as reported [16]

Three italics marked SPs (Bpr, Pel, YwfM) with their corresponding extracellular cutinase activities in both secretion hosts are discussed in the text, cf. also Fig. 4

### Consequences for optimized secretory production of heterologous proteins

The optimal combination of a target protein and a specific SP has to be determined for optimal secretion efficiency. The best combination of SP and target protein seems to be no universal feature, but is itself specific for different secretion hosts. As these combinations are not predictable until now, the best combination of production host, SP and target protein has to be evaluated for each new target protein from scratch. The screening of homologous and heterologous SPs together with other genetic libraries, e.g. for ribosome binding sites (RBS) [33] or promotors [34], in different expression hosts easily results in a huge number of possible constructs to test for an optimized production organism. Up to now, such optimal secretory production strain cannot be designed in its complexity in silico and thus, strain characterization needs to be performed by applying methods of higher throughput. To ensure characterization of a sufficient share of constructed variants of strains, these must be oversampled, i.e. that a number of clones x-times the library size is characterized.

Assuming that the selection process (i.e. the clone picking) can be approximated by the urn model with replacement, the probability to hit a specific clone at least once, P (X ≥ 1), can be calculated. For typical library sizes (n ≥ 100), this probability depends approximately only on the oversampling and is equal to ~0.95 and ~0.98 for threefold and fourfold oversampling, respectively (cf. Additional file 1 for details). Degering et al. performed a fourfold oversampling of an SP library consisting of ~400 SPs, resulting in ~1800 single clone characterizations [18]. Especially when screening genetic libraries with a high number of items (or combinations of libraries), a tradeoff between experimental workload and characterization of a sufficient library share is needed due to practical reasons.

The findings of the study show that specific SPs cause different secretion efficiencies for the same model enzyme (cutinase) when using *B.* *subtilis* [16] or *C.* *glutamicum* (this study) as host. Previous studies showed that specific SPs show comparable or highly comparable secretion efficiencies for the same model enzyme (subtilisin BPN’) when using *B.* *subtilis* and *B.* *licheniformis* or two *B.* *licheniformis* strains as secretion hosts, respectively [18]. This indicates that the phylogenetic distance of expressions hosts correlates with these observations and that the “expression environment” of the host cells is most likely the more similar, the closer hosts are related.

For an optimal combination of SP and target protein, a balanced secretion is assumed, which is an optimal interplay during all stages of protein biosynthesis and protein secretion. This includes stability of the mRNA transcript, as well as recognition of the SP by SRP after translation. The latter may be affected by the respective interaction of the SP with the target protein of choice [35], as well as insertion of the nascent polypeptide chain into the SecYEG translocation pore by SecA. Here, translational speed profile of the polypetide chain, beginning with the SP sequence, may also be important. Due to considerably increasing discrepancy in codon usage from low-GC firmicutes *B.* *licheniformis* over *B.* *subtilis* to high-GC actinobacterium *C.* *glutamicum* [36], such translational speed profile of a specific SP-protein fusion can be assumed to differ accordingly. The interplay between codon usage, translational speed and secretion efficiency has been reviewed for *E.* *coli* [37].

Post-translocational steps until the proteins’ arrival in the extracellular space can also be hampered due to a suboptimal combination of SP and target protein, causing a reduced overall secretion efficiency. For example, processing kinetics of SP could be affected by lowered affinity and/or accessibility of the cleavage site which results in blocking the translocation pore. On the other hand, this could also be caused by overloading of SPase due to highly efficient insertion of the polypeptide into the translocon. Shortage of extra cytosolic catalysts like PrsA [38] caused by low affinity to the heterologous protein or an intrinsic low folding efficiency of the heterologous protein itself may additionally reduce overall secretion efficacy.

The overexpression of genes coding for components involved in protein secretion to improve recombinant protein secretion was also reported in a recent study, using *B.* *subtilis* as expression host [39]. It was seen that overexpression of *prsA* greatly enhanced secretion performance compared to other components being overexpressed, which is consistent with previous findings [38]. The combinatorial overproduction of *prsA* and *dnaK* operon was reported to be most beneficial for two model amylases [39]. Additionally, increased copy numbers of *secD* and *secF* were shown to compensate for reduced periplasmatic secretion caused by defective SPs in *E.* *coli* [40]. These studies indicate that secretion performance of a SP identified during a SP library screening could be further enhanced by overproduction of components involved in the secretion of the target protein.

In conclusion, the overall secretion process is a carefully balanced multi-stage process, and nature has developed a species-specific toolbox of SPs, which contains an optimally designed SP for each endogenous protein to be secreted. The introduction of heterologous proteins into this machinery by biotechnologists can obviously disturb this well balanced secretion machinery. By varying the SP for secretion of a desired heterologous protein, the secretion can be re-balanced, indicated by the wide range of secretion efficiencies that is routinely observed in SP library screening studies.

## Conclusion

A library of Sec SPs from *B.* *subtilis* was successfully applied to optimize protein secretion in *C.* *glutamicum* on the example of cutinase from *Fusarium solani pisi* as model protein. After successfully transferring the whole SP library into *C.* *glutamicum*, obtained clones were characterized for SP impact on cutinase secretion. This was made using a Mini-Pilot-Plant consisting of a microbioreactor system integrated into a liquid handling robot equipped with a centrifuge for cell separation and a microplate reader for determination of cutinase activity. A wide range of activities was found depending on the SP to drive cutinase secretion, indicating that the SP library from low-GC firmicutes *B.* *subtilis* is fully functional in distantly related high-GC actinobacterium *C.* *glutamicum*.

The results were compared to a previous study, where the same SP library was used for cutinase secretion in *B.* *subtilis*. It was found that relative efficiencies of SPs with respect to extracellular cutinase activity differed dramatically between *B.* *subtilis* and *C.* *glutamicum*. Some SP showed diametrical performance in both hosts, whereas other SPs showed comparable performance in one host but not in the other host. That means a “good” SP for secretion of a target protein may completely fail to secrete in different hosts. At first glance, the results found here contrast previous work where similar performances of SPs for secretion of subtilisin BPN’ were observed in different *Bacillus* species [18], but when taking into account the phylogenetic distance of *Bacillus* sp. and *C.* *glutamicum*, different study outcomes can be explained.

Consequently, once a SP library was screened for optimal secretion performance of a certain target protein, screening results may completely differ when repeated in another expression host, especially when comparing SP library screening results between distantly related strains. Furthermore, such work-intensive screening processes can only be conducted in a feasible manner using automated devices for cultivation and wet-lab analysis designed for higher throughput. Clearly, workload becomes even more extensive when the determination of the target protein cannot rely on simple methods like chromogenic of fluorogenic activity assays, which are easily to parallelize.

To date, it is not possible to predict an optimal combination of SP, target protein and expression strain. Consequently, SP library screening to optimize protein secretion using methods of higher throughput is an important step in expression engineering and thus, recognized to be crucial for the development of a production process for technical bulk enzymes.

## Additional file

**Additional file 1.** Supporting information.

## Abbreviations

MPP: Mini-Pilot-Plant
Sec: general secretion pathway
SP: signal peptide
SRP: signal recognition particle
