# Supplementary material for: Use of a Sec signal peptide library from Bacillus subtilis for the optimization of cutinase secretion in Corynebacterium glutamicum
Source: Microb Cell Fact. 2016 Dec 7;15:208. doi: 10.1186/s12934-016-0604-6 (PMC5142396; doi:10.1186/s12934-016-0604-6)
Supplement: Supplementary file 1 — Additional file 1. Supporting information. [file 12934_2016_604_MOESM1_ESM.pdf]

Supplementary material for research article

Use of a Sec signal peptide library from *Bacillus subtilis* for the optimization of cutinase secretion in *Corynebacterium glutamicum*

Johannes Hemmerich[1,3], Peter Rohe[1,#1], Britta Kleine[1,#2], Sarah Jurischka[1,3], Wolfgang Wiechert[1,3], Roland Freudl[1,3], and Marco Oldiges[1,2,3,*]

[1]: Forschungszentrum Jülich, Institute of Bio- and Geosciences - Biotechnology (IBG-1), Jülich, Germany; [2]: RWTH Aachen University, Institute of Biotechnology, Aachen, Germany; [3]: Bioeconomy Science Center (BioSC); [#1]: Boehringer Ingelheim Pharma GmbH & Co. KG (Current affiliation); [#2]: Thermo Fisher Scientific GENEART GmbH (Current affiliation); *: Correspondence: m.oldiges@fz-juelich.de

Probability to find an item of a screened library at least once: urn model with replacement

To find a tradeoff between experimental workload and characterization of a sufficient share of possible genetic phenotypes resulting from library transformation, oversampling is suitable. This means, the number of clones tested is x-fold higher that the number of library items, i.e. the number of signal peptides (SPs). The more different genetic libraries (SPs, ribosome binding sites, promotors, etc.) are screened, the more possible combinations, represented by different clones, need to be characterized. Therefore, a probability to hit at least once such a specific combination, i.e. clone, may help to decide which amount of oversampling is conducted. This process can be approximated by the idealized urn model with replacement. Following this, the probability to hit a specific clone at least once, $P(X \geq 1)$, can be calculated according to (1), depending on the number of possible combinations (library size, denoted as n) and the x-fold oversampling (x-times the library size, denoted as m).

$$P(X \geq 1) = 1 - (1-p)^{m \cdot n}, \quad p = \frac{1}{n} \tag{1}$$

The number of balls in the idealized urn model is represented by the number of clones after transformation and the sampling of balls from the urn is represented by the clone picking procedure. It is furthermore assumed that each SP has the same probability to be transformed. However, it is impossible to identify the total number of cells that were transformed during the electroporation procedure, and this number can be easily increased by increasing the amount of competent cells and plasmid DNA during the electroporation. Therefore, it is reasonable to assume the number of transformed cells to be much higher than the number of selected colonies.

For typical library sizes ($n > 100$), this probability depends approximately only on the oversampling, see (2), and is equal to ~0.95 and ~0.98 for 3-fold and 4-fold oversampling, respectively.

$$\lim_{n \to \infty} 1 - \left(1 - \frac{1}{n}\right)^{m \cdot n} = 1 - e^{-m} \tag{2}$$

Probability to find an item of a screened library multiple times: Bernoulli process

Practically, some library items are found several times more than once during a screening process. In this study, during the screening process most SP have been identified once, but a few occurred twice, three times or even four times. One may raise the question about the expected occurrence of the multiple identification of library items (i.e., signal peptides in this study). Assuming that SPs had the same probability to be transferred, the screening process can be interpreted as a Bernoulli process, according to (3). Here, k denotes the number of k-times hitting a SP, i denotes the number of actually screened clones, and p denotes the probability as introduced above with n denoting the library size, i.e. the number of SPs. Consequently, the probabilities to identify a SP once, twice, three times or four times is calculated to be 0.293, 0.067, 0.010 or 0.001, respectively.

$$P(X = k) = \binom{i}{k} \cdot p^k \cdot (1 - p)^{i-k} \quad (3)$$

Simulating the SP library screening procedure and comparison with empirical results

An auxiliary simulation study was performed to assess the obtained occurrences of SPs regarding the assumption of complete and unbiased transfer of all 148 SPs from *B. subtilis* to *C. glutamicum*. Therefore, the formation of 250 single colonies after transforming pXMJ9-SP^{Lib}-cutinase was assumed, whereas each colony was assigned one of the SPs with the same probability. Afterwards, 66 out of these 250 colonies were sampled without replacement, and the number of SPs found multiple times (once, twice, three times, etc.) was counted. This procedure was conducted in 5000 repetitions and summarized results are shown in Figure S1, along with the experimentally determined multiple SP occurrences.

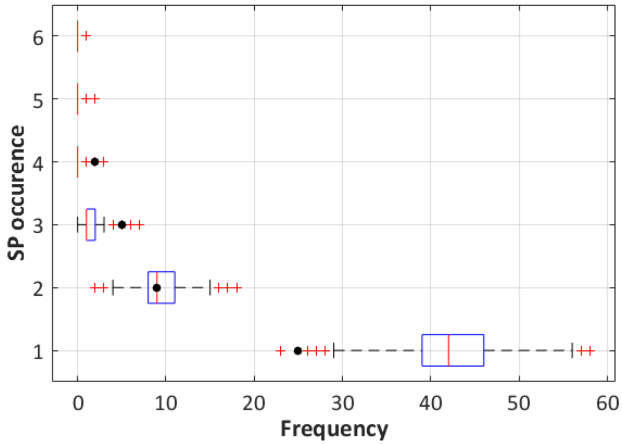

Figure S1: Box plot showing results from simulation study for the multiple occurrences of SPs. Black dots represent experimentally determined occurrences of SPs that have been determined once, twice, three times or four times.

Supplementary table

Table S1: Detailed results for cutinase secretion phenotypes from randomly selected *C. glutamicum* expression clones with undetermined SP for pEKEX2-based cutinase secretion.

#	Activity [U/mL]	#	Activity [U/mL]	#	Activity [U/mL]
1	1.66 ± 0.12	17	0.37 ± 0.09	33	0.11 ± 0.02
2	1.65 ± 0.05	18	0.29 ± 0.08	34	0.08 ± 0.04
3	1.63 ± 0.14	19	0.29 ± 0.08	35	0.08 ± 0.02
4	1.62 ± 0.10	20	0.29 ± 0.04	36	0.04 ± 0.02
5	1.07 ± 0.15	21	0.28 ± 0.02	37	0.04 ± 0.02
6	1.06 ± 0.08	22	0.27 ± 0.03	38	0.04 ± 0.00
7	1.05 ± 0.07	23	0.27 ± 0.06	39	0.04 ± 0.00
8	1.04 ± 0.04	24	0.26 ± 0.01	40	0.04 ± 0.01
9	1.04 ± 0.04	25	0.26 ± 0.07	41	0.04 ± 0.01
10	1.04 ± 0.03	26	0.25 ± 0.05	42	0.04 ± 0.00
11	1.00 ± 0.05	27	0.25 ± 0.12	43	0.03 ± 0.01
12	1.00 ± 0.08	28	0.25 ± 0.03	44	0.03 ± 0.00
13	0.39 ± 0.05	29	0.25 ± 0.02	45	0.03 ± 0.00
14	0.39 ± 0.05	30	0.23 ± 0.07	46	0.03 ± 0.00
15	0.38 ± 0.09	31	0.22 ± 0.05	47	0.03 ± 0.00
16	0.37 ± 0.09	32	0.13 ± 0.02	48	0.02 ± 0.00
